# Regeneration of tree species after 11 years of canopy gap creation and deer exclusion in a warm temperate broad-leaved forest over-browsed by sika deer

**DOI:** 10.7717/peerj.14210

**Published:** 2022-11-01

**Authors:** Jeffery, Meng Ann Ang, Dai Kusumoto, Mitsukazu Mitsugi, Maki Suzuki

**Affiliations:** 1 Graduate School of Frontier Sciences, The University of Tokyo, Kashiwa City, Chiba Prefecture, Japan; 2 University of Tokyo Chiba Forest, Graduate School of Agricultural and Life Sciences, The University of Tokyo, Kamogawa City, Chiba Prefecture, Japan

**Keywords:** Tree regeneration, Deer overpopulation, Deer exclusion, Gap creation

## Abstract

Deer overpopulation is a major threat to forest ecosystems worldwide resulting in loss of natural vegetation cover and increased sapling mortality. To resolve this problem of deer overpopulation, different strategies such as deer exclusion and gap creation have been explored to determine more efficient methods to restore deer-damaged forest ecosystems. In the current study, we applied a 2 × 2 factorial design of four different treatment groups in warm temperate secondary forests: closed canopy with deer as control, closed canopy without deer, clearcut with deer and clearcut without deer. We compared the decadal change in tree foliar cover and tree species richness among treatment groups to assess tree regeneration success. We also selected six tree species (*Abies firma, Quercus acuta, Eurya japonica, Cinnamomum tenuifolium*,* Castanopsis sieboldii* and *Neolitsea sericea*) that are common in the studied region and compared their regeneration success among the treatment groups. In the absence of deer, clearcutting increased the diversity of tree species and accelerated sapling growth, while under closed canopy conditions sapling heights did not exceed two meters. Tree saplings tended to be less abundant in treatments with deer compared to their counterpart, suggesting limited successful recruitment of saplings at the current deer density (10–13.5 deer km^−2^). In clearcut-with-deer treatment, non-tree species became abundant, and negatively affected recruitment of tree species as was suggested by regression analysis. However, these general trends were not equal for all tree species. Although clearcut-without-deer treatment facilitated sapling recruitment of all six tree species, *Q. acuta, C. tenuifolium* and* C. sieboldii* required deer exclusion for sapling recruitment while *A. firma, N. sericea* and *E. japonica* required increased light availability. Consequently, informed decisions can be made by identifying whether certain tree species are capable of naturally recruiting without human intervention and how best to ensure successful recruitment if necessary. By implementing effective strategies, time and resources will be saved, and management goals such as reestablishing tree cover rapidly and increasing tree species diversity can be achieved.

## Introduction

Deer overpopulation is a major concern in many countries across the globe, associated with suppressed tree regeneration and increased damage to forest ecosystems (Gill, 1992; Tremblay, Huot & Potvin, 2007; Koda et al., 2008). Deer browsing increases the mortality of tree saplings, preventing the successful establishment of new generations (Coomes et al., 2003; Takatsuki & Hirabuki, 1997; Takatsuki, 2009), with negative implications for the regeneration of forest ecosystems, highlighting the importance of effective forest restoration methods (Castro et al., 2021). In addition, deer browsing can be selective and this biased herbivory can affect plant species richness either positively (through suppression of a strong competitor) or negatively (increased mortality by direct browsing) (Gill, 1992; Côté et al., 2004; Takatsuki, 2009). In cases when the negative effects of deer on vegetation are not negligible, the response is to decrease the deer population density. However, even at low population densities, deer have been known to interrupt plant regeneration and prevent the recovery of original vegetation (Coomes et al., 2003). If that occurs, temporary and complete exclusion of deer from the target area using deer exclosure fences can support vegetation recovery. The effectiveness of deer exclusion fencing is, however, dependent on the local environmental conditions such as light availability (Tremblay, Huot & Potvin, 2007; Suzuki, 2013). It is necessary to assess the effectiveness of deer exclusion fencing in promoting tree regeneration and recovery of damaged forest ecosystems under various environmental conditions.

Light availability is an important environmental factor that causes complex responses in the plant community. Plant regeneration can be promoted by improving the light availability through artificial gap creation (Tremblay, Huot & Potvin, 2007; Suzuki, 2013). Gap creation tends to enhance the positive effects of deer exclusion fencing on plant species richness (Suzuki, 2013; Sabo et al., 2019), survival and growth of tree saplings (Shimoda et al., 1994; Tremblay, Huot & Potvin, 2007; Tamura & Nakajima, 2017; Nagashima, Shimomura & Tanaka, 2019; Petersson et al., 2020). According to these experiments, gap creation tends to speedup forest regeneration compared to simple deer fencing under closed canopies. However, it is also suggested that the regenerated vegetation is often dominated by light-demanding species such as ferns (Takatsuki, 2009), disturbance related species such as *Eurya japonica* (Manabe & Yamamoto, 1997) or herbivory-tolerant ones (Gill, 1992; Côté et al., 2004; Royo & Carson, 2006), being different from that before gap creation. Furthermore, when fast-growing competitive plants are present, gap creation may result in increased competition (Kern et al., 2012) and the formation of “recalcitrant layers” that inhibit regeneration of tree species (Royo & Carson, 2006). If the management goal were to promote regeneration of the current tree flora, deer exclusion under closed canopy conditions could be a safer option, though it may be more time-consuming.

Currently, most studies investigating the combined treatment effects of deer exclusion fencing and gap creation on forest regeneration have short experimental periods (Shimoda et al., 1994; Nagashima, Shimomura & Tanaka, 2019; Tremblay, Huot & Potvin, 2007; Suzuki, 2013; Suzuki & Ito, 2014; Sabo et al., 2019; Suzuki et al., 2021), with few studies exceeding 10 years (Tamura & Nakajima, 2017). The general outcome of 10-years treatments is that the fencing of large canopy gaps (400 m^2^, (Suzuki et al., 2021) successfully facilitated forest regeneration, ensuring the survival of tree saplings (Tamura & Nakajima, 2017). In contrast, gap creation in the presence of deer increased mortality of tree saplings (Tamura & Nakajima, 2017) and consequently, facilitated establishment of short bushes of non-tree species (Suzuki et al., 2021). These preceding studies suggest benefits of combined treatments for the regeneration of forests. However, the benefits of the combined treatment may not be evenly shared by all tree species. For example, *Abies firma* (Tamura & Nakajima, 2017) and evergreen *Quercus* species (Tsujino & Yumoto, 2004), both shade-tolerant and browsed by deer, showed higher sapling abundance under closed canopy conditions compared to gap plots (Tamura & Nakajima, 2017; Petersson et al., 2020). The combined treatments may not be optimal for these species, possibly due to their slow growth rates and high inter-specific competition within gaps. Other tree species such as *Eurya japonica* and *Neolitsea sericea* showed positive response to deer exclusion (Tsujino & Yumoto, 2004), but considering their relatively high tolerance to moderate deer densities (Suda, Araki & Maruyama, 2001), these species might show higher survival rates and competitive advantage outside the deer fences. These specie-specific response to light conditions and herbivory by deer would affect the forest succession and species composition of each treatment. So far, monitoring records of more than a decade are too limited to clarify the pros and cons of each treatment.

In the current study, we evaluated the 11-years effects of the combined treatments on the regeneration of selected common evergreen tree species in warm-temperate broadleaved forests. The current study focused on tree species, which forms the canopy and subcanopy layers of mature forests. The forests in the Boso peninsula were selected taking advantage of the established experimental treatments which have been maintained since 2008. The early changes in the ground vegetation cover (both woody and non-woody species below 2 m height) and species richness up to two years after gap creation (2007–2009) and those up to 7.5 years (2007–2015) have been described by previous studies (Suzuki, 2013; Suzuki et al., 2021) in the same study sites. These studies used a research scheme for ground vegetation sampling (five replications of 1 × 1 m quadrats). Instead, the current study used sampling areas larger by a magnitude of five (5 × 5 m quadrats) and successfully evaluated the treatment effects on tree regeneration which was not addressed in previous studies. We explored the following questions: (1) Did a decade of deer presence after clearcut affect the foliar cover and species composition of trees? (2) In the event of a clearcut, did competition with non-tree species prevent tree regeneration? (3) What was the response of common tree species in the current study towards the experimental treatments? Understanding these questions would improve our understanding of forest regeneration and succession, as well as provide forest managers with more management options for restoring forest ecosystems and increasing densities of targeted tree species.

## Materials & Methods

### Study sites

We utilized existing study plots established in three warm temperate evergreen secondary forests within the University of Tokyo Chiba Forest (UTCBF) located in the southern part of the Boso Peninsula, Japan (KBS, HRT & HNK, 35°8–11′N, 140°7–9′E) described by Suzuki (2013), see (File S1). The study sites were coppiced for fuelwood before abandonment in the 1960s (Suzuki, 2013). The study sites are located on hill slopes with angle of elevations between 28–36 degrees and are 2–5 km apart from each other. Hinokio (HNK) and Hiratsuka (HRT) are located 300 m above sea level (a.s.l), with mean, minimum, and maximum annual air temperatures of 14, −4.5, and 33.8 °C, respectively in 2019, and annual precipitation of 3,007 mm (The University of Tokyo Forests, 2021). Kotsubosawa (KBS) is located closer to the nearest coastline (2 km) compared to HNK and HRT (7 km) with an altitude of 50 m a.s.l. The mean, minimum, and maximum annual air temperatures for KBS in 2019 were 14.8, −2.3, 33.2 °C, respectively, with an annual precipitation of 2,617 mm (The University of Tokyo Forests, 2021).

Prior to the experiment, the vegetation in the study sites were mainly tall trees with sparse evergreen shrubs and vines at the forest floor. Dominant trees in the canopy layer were *Quercus glauca* and *Castanopsis sieboldii* in KBS, *Q. glauca* and *Cleyera japonica* in HNK, and *Q. acuta* in HRT. Subcanopy layer of all the three sites had *E. japonica* as a dominant species. The subcanopy layers also included other evergreen species such as *Myrsine seguinii* and *Camellia japonica* (KBS), *C. sieboldii* and *Q. myrsinifolia* (HNK), and *C. japonica* and *Dendropanax trifidus* (HRT). The ground layer of all the three sites had sparse evergreen shrubs and lianas (Suzuki, 2013).

Sika deer (*Cervus nippon*) are native to this region and their density within the UTCBF in 2018 was estimated between 10–13.5 deer km^−2^ (Hisamoto et al., 2019), exceeding the target set by the Chiba prefecture government for forest conservation, between 3–7 deer km^−2^ (Chiba Prefecture of Japan, 2017). This target was based on previous studies conducted by the prefecture government where negative effects on sapling recruitment of *Quercus* species were detected between deer densities of 5–15 deer km^−2^ (Chiba Prefecture of Japan, 2004). Efforts by the prefectural government to curb deer densities through hunting and trapping are still ongoing, though the efforts are becoming more challenging due to decreasing number of active hunters. Prior to the experiment in 2008, the vegetation at the ground layer under the closed canopy had disappeared due to deer herbivory. Thus, we considered the deer density in our study (10–13.5 deer km^−2^) to be overpopulated with a negative influence towards tree sapling regeneration.

### Experimental treatments

The experimental design within each study site (HNK, HRT and KBS) included two canopy cover levels (closed canopy and clearcut), each within about 20-by-20 m study plots (File S1). Following the clearcut treatment prior to the experiment in 2008, we also removed all vegetation and fallen woody materials from the forest floor. The gap size (ca. 400 m^2^) resulting from the clearcut was greater than the typical sizes of natural gaps in Japanese temperate forests (30–140 m^2^; Yamamoto, 2000). For this study, we used the words gap and clearcut interchangeably, both referring to study plots where vegetation had been removed. Within each study plot of about 20 by 20 m, we created four 10-by-10 m subplots. Two of the subplots were installed with polyethylene nets of 30 mm mesh up to 1.5 m height from the ground to exclude middle to large-sized mammals, while the other two subplots were open and accessible to herbivores. This therefore created four treatment types: “Closed canopy and open to herbivores” referred to as Control (Ctrl), “Closed canopy with herbivores excluded” as treatment E, “Clearcut and open to herbivores” as treatment G and “Clearcut with herbivores excluded” as treatment EG with a total of six subplots each (*n* = 24).

The vegetation in the clearcut plots reflects the recruited vegetation after 11 years with/without deer. Meanwhile, the vegetation in the closed canopy plots inseparably includes vegetation from before the experiment in 2008. Thus, the differences between the closed canopy plots (Ctrl and E) reflect changes from 11 years of deer exclusion. The clearcut plots (G and EG) and the closed canopy plots (Ctrl and E) are separated more than 10 m apart in HNK and KBS, allowing for the natural vegetation to function as a buffer between different experiments, while the clearcut and closed canopy plots were adjacent to each other in HRT due to topographic limitations. The experimental design allows for seed dispersal from the natural vegetation surrounding the study plots into the clearcut plots (see further details in Suzuki et al., 2021). Based on the tree census data in 2006 (Suzuki et al., 2010), the tree species composition (DBH >1 cm) prior to the experiment (in 2008) was not biased among the different treatments.

### Vegetation survey

To describe the overall features of vegetation in each treatment, we conducted vegetation surveys within 5 m quadrats established at the centre of each 10 m subplot to avoid boundary effects, between October and early November in 2019. The percentage of foliar cover and the maximum height of all plant species were recorded and classified according to their growth forms: trees and non-tree species (shrubs, vines, graminoids and forbs). The foliar cover of each plant species was visually estimated by assessing their percent cover within the 5 m quadrat, accounting for those in the ground and canopy layer. We took the sum of all plant species that corresponded to each evergreen tree species, deciduous tree species and non-tree species. In the case where there is high overlap of foliar cover, such as in trees, the total foliar cover could exceed 100%. The plant species with a foliar cover of less than 1% was noted as **‘+’** and assigned a value of 0.1% while those with cover exceeding 100% was assigned a value of 99.9% during statistical analyses. For this study, we defined non-tree species cover in each subplot (within the 5 m quadrat) as the sum of foliar cover of shrub, vine, graminoid and forb species.

For each treatment, we calculated tree species richness (number of different tree species found in 150 m^2^ = (5 × 5 m^2^) ×6 subplots per treatment), mean percentage cover (*MC*) of evergreen trees and *MC* of trees palatable to deer. We assessed the palatability of tree species to deer based on records of deer herbivory under varying conditions in Japan (Suzuki et al., 2008; Agetsuma, Agetsuma-Yanagihara & Takafumi, 2011; Suzuki, 2013; Hashimoto & Fujiki, 2014). In situations where the records were inconsistent, we determined the palatability to deer according to our experience of the study site.

### Tree sapling recruitment and growth

Six common evergreen tree species were selected to assess the response of their saplings to the treatments: *Abies firma, Quercus acuta, Eurya japonica, Cinnamomum tenuifolium, Castanopsis sieboldii* and *Neolitsea sericea*. All the six species were judged as fairly common based on their frequency in subplots of each treatment (*f*): they appeared in more than 3/6 plots of at least one experimental treatment, and their mean percent cover (*MC*) exceeded 10%, or they showed highest *MC* values in at least one treatment. The tree species found in less than 3/6 plots in any of the experimental treatments were avoided, because their biased occurrence might be accidental and not due to the treatment effect. Among the six tree species, only the *MC* of *Q. acuta*, *E. japonica*, *C, tenuifolium* and *C. sieboldii* exceeded 10% in at least one experimental treatment. Meanwhile in treatment G, the highest *MC* recorded was of *N. sericea* and *A. firma* exceeding 1.5%. Thus, both species were considered common species for treatment G.

We categorized the saplings of the above six species in October 2020, according to the following height classes: (i) between 0.1 m to less than 0.3 m, (ii) between 0.3 m to less than 1 m, (iii) between 1 m to 2 m and (iv) higher than 2 m. We calculated the sapling densities based on the total sapling count per unit area sampled (m^−2^). The saplings of height classes (i) and (ii) were sampled within a 5-by-1 m belt transect (5 m^2^ ×2 subplots ×3 study site = 30 m^2^) in the centre of each study plot. Class (iii) and class (iv) saplings were sampled within 5-by-5 m (150 m^2^) and 10-by-10 m quadrats (600 m^2^) respectively. Based on our observations in the closed canopy plots, there was a clear distinction between mature canopy trees that had stems with DBH >10 cm and saplings below the canopy with DBH <10 cm of the target species. The closed canopy plots were mostly devoid of ground vegetation due to long-term deer herbivory. Unlike the clearcut plots, the vegetation in the closed canopy plots were not disturbed and the differences between Ctrl and treatment E depict the effectiveness of deer exclusion fencing on sapling recruitment and survival. Thus, we only focused on target species saplings with DBH <10 cm in the closed canopy plots while in the clearcut plots, all the regenerated target species were counted and assumed as an effect of treatment.

### Statistical analyses

#### Treatment effects on selected parameters

We assessed the effects of the experimental treatments on tree species richness using Poisson regression with a log link taking treatment as the fixed variable while accounting for the effects of study site and the study plots of 20 x 20m as nested random factors. To assess treatment effects on vegetation cover, given the binomial distribution nature (non-discrete) of the data, we applied beta regression with logit link, setting 99.9% as the maximum foliar cover. We again applied beta regression to assess treatment effects on foliar cover of evergreen tree species and palatable tree species. The *glmmTMB* package for *R* was used for above Poisson and beta regressions. If the individual treatment effect was statistically significant in the regression analyses (*Wald* test, *p*-value <0.05), a *post hoc* pairwise comparison of estimated marginal means (*emmeans* command of package *emmeans* in *R*) using the Tukey-Kramer method was applied.

To compare how the tree species composition differed among the experimental treatments, we first generated dissimilarity indices (*vegdist* command of *vegan* package) based on the presence/absence data and then obtained the mean dissimilarity among treatment groups (*meandist* command of package *vegan* in *R*).

#### Relationship between Tree foliar and non-tree species cover in clearcut plots

To estimate the negative effects of the non-tree species in the clearcut plots (EG and G) onto the foliar cover of tree recruitments, we applied beta regression following a beta distribution with logit link. We again accounted for the effects of study site and the 20 ×20 m study plots as nested random factors in the regression model. Because the foliar cover of trees and non-tree species were both affected by deer (see Results), deer presence was added as a fixed variable in the model to statistically remove the effect. Multicollinearity between the explanatory variables in the model was assessed using the *performance* package in *R*, revealing low correlation between them (VIF <1.1). We assessed the goodness-of-fits of each regression model (M_0_ to M_3_, File S3) to data by drawing *Q-Q* and residual plots using *simulateResiduals* function in *DHARMa* package of *R*. The model for tree species cover explained by deer and non-tree species cover showed significant heteroscedasticity, and thus the issue was solved by removing the random effect of subplots which resulted in the best fit model based on AIC and BIC values. We then plotted the best fit model to estimate how tree foliar cover changed with increasing non-tree species cover, with and without deer.

#### Treatment effect on sapling height and density of target tree species

To examine the treatment effects on sapling height of target species, we performed non-parametric *Kruskal-Wallis* rank sum test (*Kruskal.test* function in *R*) on the treatment effect on sapling height class (i–iv) for each species, given the non-normal distribution of the data. If the treatment effect was significant (*p*-value <0.05) a *post hoc* test was applied to determine significant differences between pairwise treatments. For the *post hoc* test, we used *Dunn’s* test (*dunn.test* function in *dunn.test* package in *R*) with *Bonferroni* adjustment. The total sapling density of each target species per subplot was calculated as the sum of height class i to iv sapling densities. We again applied *Dunn’s* test with *Bonferroni* adjustment to determine significant treatment effects (pairwise *p*-value <0.05) on total sapling density for each target species. The residual diagnostics of the above regression models were verified using *DHARMa* package. These processes were implemented using the statistical software *R* (R Core Team, 2021).

## Results

### Plant foliar cover

**Figure 1:**
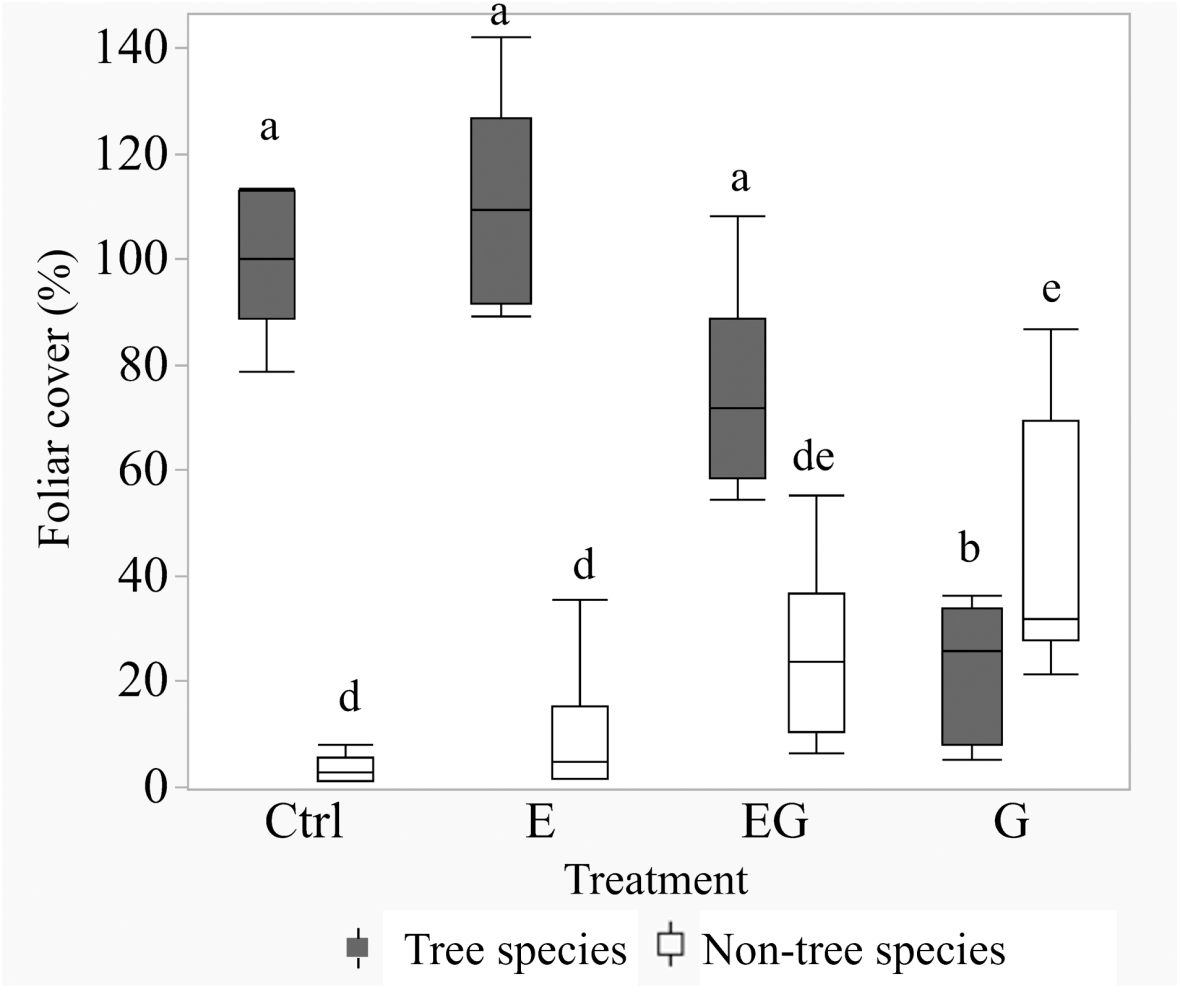
Boxplot of tree and non-tree species cover in the 5 m quadrats in 2019. Tree cover in the Ctrl and treatment E represents changes after deer exclusion while tree cover in treatment G and EG represents the regenerated tree cover after 11 years. The non-tree species cover is the calculated sum of graminoid, forb, shrub and vine cover (non-tree species). Significant differences following *post hoc* analysis of pairwise treatments are displayed by different lowercase letters; ‘a-b’ for tree cover and ‘d-e’ for non-tree species cover.

**Figure 2:**
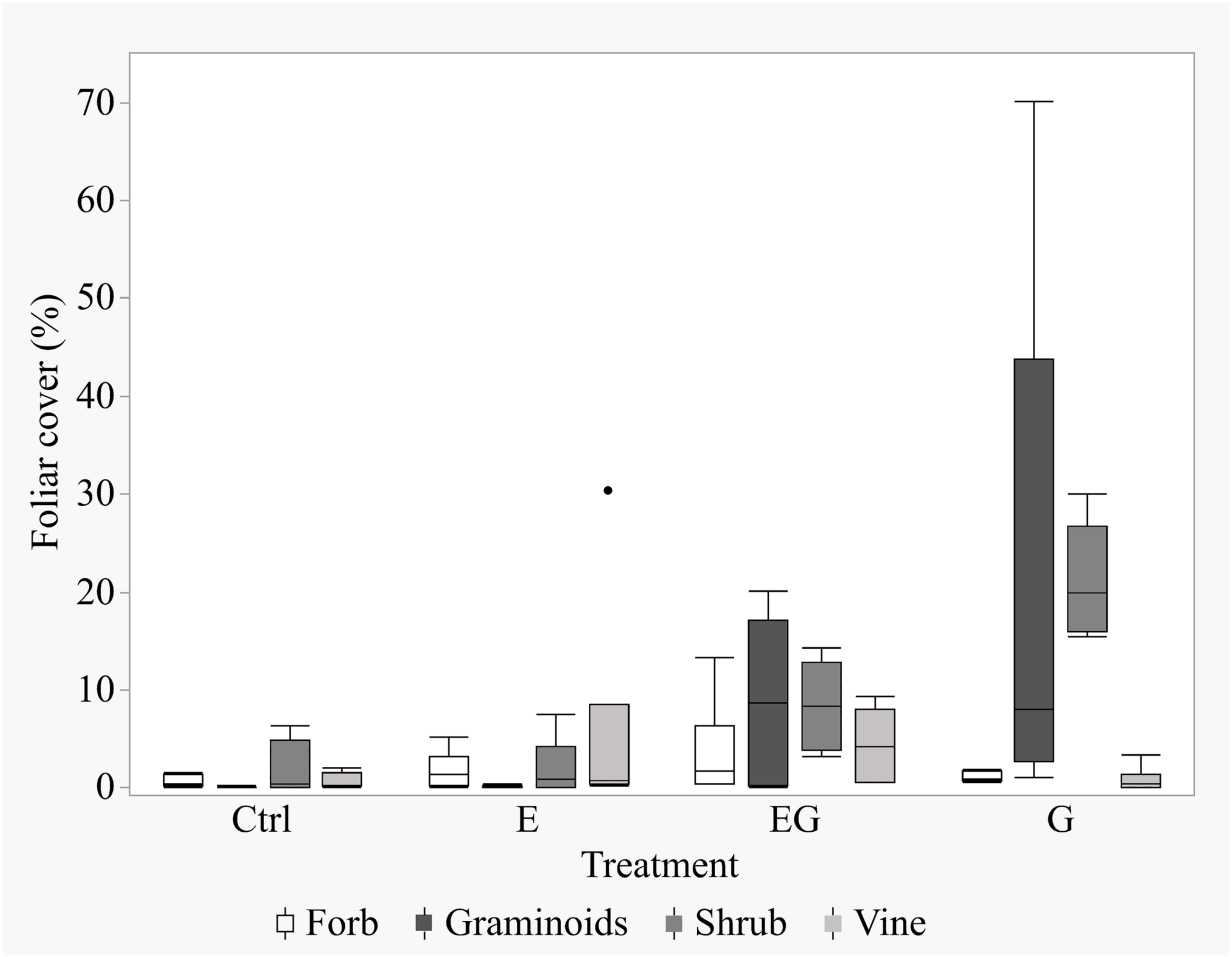
Boxplot of non-tree species cover in the 5 m quadrats in 2019. The non-tree species were categorized according to their growth forms within the 5m-by-5m vegetation sampling quadrats for each treatment. The percent foliar cover data is displayed in the order of Forbs, Graminoids, Shrubs and Vines for each respective treatment.

After 11 years of treatment, the non-tree species cover remained depauperate in the closed canopy plots (Ctrl and E) while it increased in the clearcut plots (G and EG, Fig. 1). Deer exclusion increased the foliar cover of trees, forbs and vine species (EG >G and E >Ctrl) while graminoid and shrub cover increased in the clearcut plots (G >EG, Fig. 2). The treatment EG plots were dominated by tree species, similar to the closed canopy plots whereas treatment G plots were dominated by non-tree species. Foliar cover of the recruited trees in treatment EG was significantly higher than in treatment G (Fig. 1). Tree cover in treatment EG was similar to that in closed canopy plots, while the tree cover in treatment G was significantly lower. Foliar cover of non-tree species was highest in treatment G, significantly higher than the closed canopy plots (Fig. 1). The foliar cover of non-tree species in the treatment G plots showed high spatial variation between and within study sites. For example, the non-tree species cover in both treatment G plots in KBS were >60% and approximately 30% respectively while in HNK, one plot was >85% and the other was approximately 21%. In addition, the dominant plant form for the non-tree species in treatment G plots varied greatly, with either graminoid, shrub or both forms being dominant.

### Tree species composition

We recorded 24 tree species (mature + regenerated) among the Ctrl subplots with similar values in treatment E and G (Table 1). Treatment EG had the highest number of tree species (45 species), and its mean tree species richness per subplot was significantly higher among the experimental treatments. Treatment EG had significantly higher cover of evergreen and palatable tree species compared to treatment G. At the same time, foliar cover of evergreen species and palatable tree species that regenerated in the clearcut plots (G, EG) were both still lower compared to the closed canopy plots (Ctrl, E).

**Table 1:** Tree species richness (including mature trees in closed canopy plots) and mean percent cover (*MC*, %) of evergreen and palatable trees among subplots of each treatment group in 2019. Significant differences following *post hoc* analysis of pairwise treatments are displayed as different lowercase letters (a–c) in superscript.

Treatment		Tree species richness				Tree foliar cover (%)				
						Evergreen			Palatable	
		Total (150 m^2^)	Mean	SD		*MC*	SD		*MC*	SD
Ctrl		24	8.8^a^	2.9		96.0^a^	14.4		99.2^a^	13.6
E		24	9.7^a^	2.7		104.2^a^	26.7		105.1^a^	27.7
G		23	8.7^a^	3.0		12.9^b^	12.2		5.4^b^	6.5
EG		45	17.3^b^	4.5		49.0^c^	17.8		71.3^a^	16.2

The composition of regenerated tree species was different between the closed canopy and gap plots, showing a difference of at least 76% (Table 2). Tree composition in the gap plots between treatment G and EG was also highly different (78%). Among the experimental treatments, treatment G showed the highest difference in tree composition from the other treatments. The most abundant species (based on *f* ≥ 3 and greatest *MC*) within treatment plots was also different: *Q. acuta* and *C. sieboldii* in Ctrl and treatment E plots, *E. japonica* in treatment EG plots and *N. sericea* in treatment G plots (Table 3).

**Table 2:** Mean dissimilarity matrix of tree species composition (presence/absence) among experimental treatments.

Treatment	Ctrl	E	G
E	0.67		
G	0.82	0.81	
EG	0.81	0.76	0.78

**Table 3:** Mean percentage cover (*MC*, %) of the target evergreen tree species and their frequency in the plots of each treatment (*f*).

Species name	Status^*^		Ctrl			E			G			EG	
	PA		*MC*	*f*		*MC*	*f*		*MC*	*f*		*MC*	*f*
*Abies firma*	N		+	3		+	3		1.8	5		+	4
*Neolitsea sericea*	N		+	3		+	3		2.9	3		+	3
*Castanopsis sieboldii*	P		25.2	4		33.7	5					2.7	1
*Eurya japonica*	P		17.8	6		9.3	6		+	6		16.7	6
*Cinnamomum tenuifolium*	P		11.8	4		3.4	6		1.3	1		2.5	4
*Quercus acuta*	P		25.5	5		30.5	6					3.8	4

**Notes.**

* Palatability to deer (PA: P alatable or N on-palatable) were based on Suzuki et al., 2008; Agetsuma, Agetsuma-Yanagihara & Takafumi, 2011; Suzuki, 2013; Hashimoto & Fujiki, 2014. Species with MC less than 1% are displayed as ‘+’.

**Figure 3:**
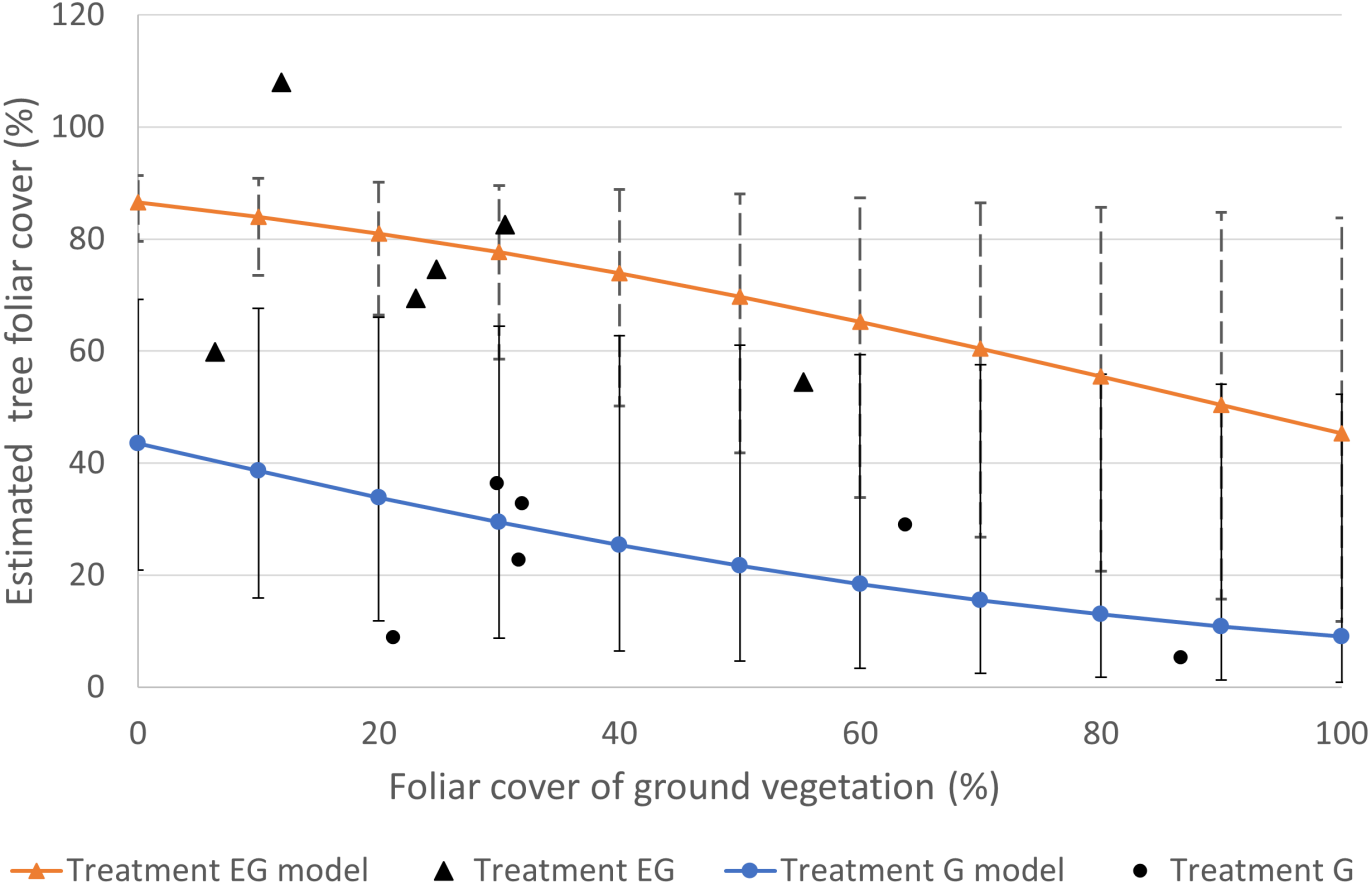
Plot of estimated tree foliar cover (%) against non-tree species cover (%) based the best fit regression model (File S3). The individual data in the study plots are displayed as single data points. The error bars represent the standard error of the estimated tree foliar cover.

The foliar cover of the trees recruited in the clearcut plots showed negative responses to the foliar cover of non-tree species and deer presence (File S3). The highest estimated tree foliar cover (86.5%) was in the absence of deer and non-tree species while the least tree cover (9%) was in the presence of both deer and dense (100%) non-tree species cover (Fig. 3). According to the beta regression model, as the non-tree species cover increases, the suppression effect on the tree foliar cover also increases. The non-tree species cover in the current study was greater in treatment G (21–87%) compared to EG (6–55%). At 50% non-tree species cover, the estimated tree foliar cover loss was 19.4% ((86.5–69.7)/86.5) in treatment EG while the loss in treatment G was approximately 50% ((43.5–21.7)/43.5). The current analysis successfully shows the presence of both direct and indirect negative effects of deer on tree regeneration. Overall, the combined effects of deer and dense non-tree species cover in treatment G severely limited regeneration of tree foliar cover.

### Sapling abundance of common tree species

**Figure 4:**
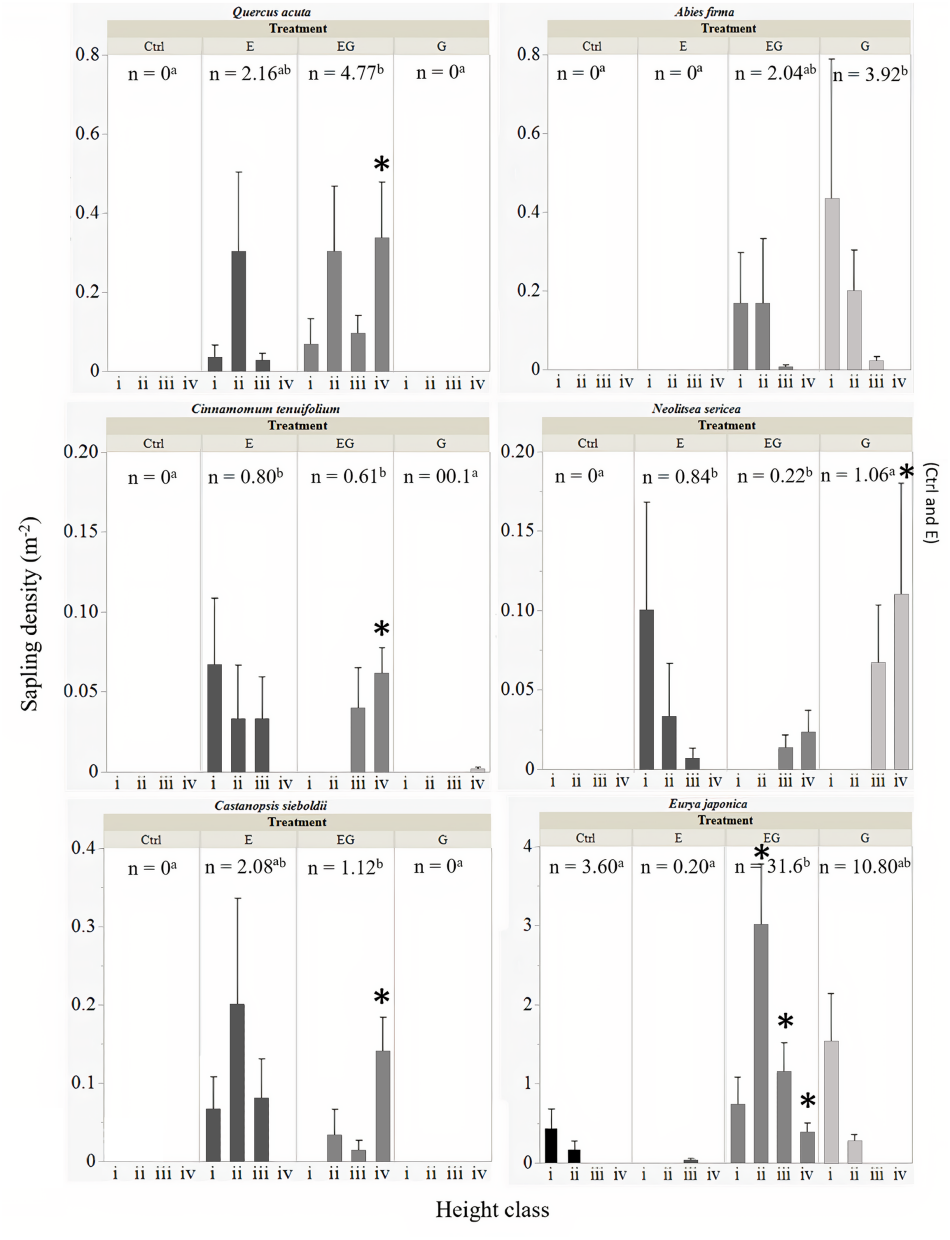
Total sapling density (n) of common tree species among experimental treatments. The error bars represent the standard error of the mean sapling density per sapling height class: (i) 0.1 m ≤ *x* < 0.3 m, (ii) 0.3 m ≤*x* < 1 m, (iii) 1 m ≤ x ≤ 2 m and (iv) > 2 m. The significant differences between total sapling densities (n) among treatments is displayed by different letters in superscript. Significant difference of sapling counts among treatments within each respective sapling height class per target species is displayed by an asterisk.

All six common tree species were able to survive and regenerate in treatment EG, whereas in treatment G, only *A. firma*, *E. japonica* and *N. sericea* regenerated (Fig. 4). The saplings of *Q. acuta*, *C. tenuifolium* and *C. sieboldii* were only observed in treatments with deer exclusion fencing (E and EG, Fig. 4). The densities of small saplings (height classes i –iii) of these three species (except for *Q. acuta*) tended to be slightly higher in treatment E compared to EG while large saplings (height class iv) of all three species were significantly higher in treatment EG compared to the other treatments. The cut stumps of *Q. acuta* tend to sprout many adventitious buds. However, based on our observations in the field, no sprouts were observed in the presence of deer, though mature trees higher than two meters that exceeded the reach of deer were observed in Ctrl plots outside the 5 ×5 m quadrats (Suzuki M; Ang JAM, 2019-2021, pers. obs). The total sapling density of *Q. acuta* was significantly higher in treatment EG compared to the other treatments.

The sapling recruitment of *C. tenuifolium* showed similar trends to *Q. acuta*, though the overall sapling density was relatively lower than *Q. acuta* (Fig. 4). The highest total sapling density was observed in treatment E compared to unfenced plots (Ctrl and G). Saplings of *C. tenuifolium* less than one meter height were not detected within treatment EG while saplings greater than two meters height were absent from treatment E, suggesting the absence of younger saplings in EG.

The sapling densities of *C. sieboldii* were relatively higher in treatment E compared to EG and unfenced plots (Fig. 4). Like *C. tenuifolium*, saplings of all height class i to iii were observed in treatment E while young saplings below thirty centimeters height were absent from treatment EG.

The saplings of *A. firma* were mainly recorded in the clearcut plots (G and EG), showing an L-shaped population size structure with increasing height class (Fig. 4). Between the gap plots, sapling densities were higher in treatment G compared to EG, though no significant differences were detected.

Interestingly, *N. sericea* showed regeneration under three different treatment conditions (Fig. 4). Saplings of less than one meter in height were only recorded in treatment E, showing an L-shaped population size structure which greatly differed from saplings in treatment EG and G. In the gap plots, saplings under one meter height were not detected, though trees greater than one meter height were present. The highest sapling density for this species was observed in treatment G but no significant differences were detected among treatments.

The highest sapling density in this study was observed for *E. japonica* where the saplings increased greatly in the gap plots, significantly higher in treatment EG compared to the other treatments (Fig. 4). Saplings of *E. japonica* showed an L-shaped population size structure even in the presence of deer, reflecting a high establishment rate from plentiful seeds. In the presence of deer, the regeneration of *E. japonica* saplings decreased, and the sapling heights were suppressed to less than one meter.

## Discussion

### Regeneration of vegetation after canopy gap creation

After 11 years, the vegetation successfully regenerated in the experimental treatment plots, though the ground vegetation cover (<2 m height) in the closed canopy plots remained low. The current study shows similar trends in vegetation cover to previous short-term studies in the same study plots (Suzuki, 2013; Suzuki & Ito, 2014; Suzuki et al., 2021): 1. Gap creation increased abundance of non-tree species; 2. The maximum height of tree species was greater in treatment EG compared to G; and 3. Non-tree species were the dominant vegetation in treatment G plots whereas trees were dominant in treatment EG plots. However, the previous studies did not address the prominent issue of tree species regeneration among the study plots. The current study instead revisits the same study site after a decade since the experiment began and compares tree foliar cover and sapling densities of target tree species among the treatment groups. Additionally, treatment E plots, which had shown almost no difference from Ctrl plots in previous studies, had higher cover of non-tree species relative to Ctrl plots in the present study.

In treatment G, the dominance of non-tree species persisted even after 11 years, following the earlier trends reported by short-term studies (Suzuki, 2013; Suzuki & Ito, 2014; Suzuki et al., 2021). Direct deer browsing and indirect trampling likely increased tree sapling mortality and consequently their regeneration failure (Gill, 1992; Coomes et al., 2003; Côté et al., 2004; Tremblay, Huot & Potvin, 2007; Koda et al., 2008; Takatsuki, 2009). Gap creation showed positive effects towards foliar cover and species richness of trees only when deer were excluded, corresponding with our research question (1). This result was consistent with past literatures with short study periods (Shimoda et al., 1994; Tremblay, Huot & Potvin, 2007; Nagashima, Shimomura & Tanaka, 2019; Sabo et al., 2019; Petersson et al., 2020).

During the early phase of regeneration after gap creation, tree saplings would have been in competition with non-tree species and deer (Kern et al., 2012), providing insight to our research question (2). Our results suggest that the negative effects of non-tree species and deer to be additive, resulting in stronger suppression effects when both variables are present in G plots. The lone effect of non-tree species in EG plots did not appear to strongly inhibit tree regeneration compared to G plots. Based on the regenerated tree cover in treatments G and EG, the suppression effect by deer appears to be greater than that of non-tree species. Deer herbivory on tree saplings (particularly palatable species) would negatively affect growth and mortality of saplings; freeing up nutrients and space (or light) for non-palatable plants, such as ferns, to become dominant. Furthermore, the higher non-tree species cover in treatment G compared to EG suggests a greater chance of formation of “recalcitrant layers” that inhibit regeneration of tree species (Royo & Carson, 2006) appearing in treatment G. We caution the interpretation of the regression model outside of our actual data, due to our small sample size; nonetheless, our results indicate a higher rate of tree regeneration and species richness in treatment EG relative to treatment G.

Meanwhile, the differences in tree species composition based on mean dissimilarity between G and EG plots was likely a result of negative impacts from deer and non-tree species during the primary stages of regeneration, corresponding to the second part of our research question (1). Deer herbivory and subsequent increased competition from non-tree species in G plots decreased the mean species richness and foliar cover of tree species, contributing to the major difference in tree species composition between G and Ctrl plots. Although EG plots also showed major differences in tree species composition from Ctrl plots, they shared common tree species with closed canopy plots. In addition, mean foliar covers of evergreen trees and palatable tree species were both significantly higher in EG plots than in G plots. Considering these characteristics of species composition in EG plots together with the high species richness, if the management goal were to increase tree diversity, treatment EG may be a better option compared to treatments G, E and Ctrl. In contrast, if the management goal were to increase sapling abundance with the least impact to the current forest composition, treatment E may be a safer option. However, the current results only reflect the changes after 11 years, which is still a brief period in forest succession. Longer observations are necessary to confirm whether the current tree species composition persists or changes as secondary succession progresses.

### Treatment effects on the regeneration of common tree species

Although the treatment EG generally enhanced regeneration of tree species, the six common species in our study site showed characteristic behavior in response to different treatment conditions, corresponding to our research question (3).

Three common species of warm-temperate forests, *Q. acuta*, *C. siebioldii* and *C. tenuifolium*, showed similar responses to deer exclusion indicating regeneration failure under the current deer density. Our results support those of Petersson et al. (2020) where *Quercus* saplings were more abundant in deer-excluded clearcut plots compared to deer-excluded closed canopy plots. Our results show that saplings of these three species remained below 1 meter height in treatment E but were able to exceed two meters height in treatment EG, suggesting rapid growth with increased light availability. Thus, for these species, deer exclusion is necessary for their sapling recruitment. If the management goal were to maintain the current ecosystem (mature trees) while ensuring a sapling bank of these species under the closed canopy that can rapidly grow once conditions become favorable, deer exclusion may be sufficient.

On the other hand, *A. firma* and *E. japonica* showed higher sapling densities under increased light conditions (G and EG) compared to under closed canopies even in the presence of deer. This suggests that for these two species, light was the limiting factor rather than deer. Tamura & Nakajima (2017) recommended the combined treatments (EG) to support regeneration of *A. firma* under deer densities of 10–17 deer km^−2^. However, our results suggest the opposite with higher sapling densities in treatment G compared to EG. In our study, *A. firma* does not appear to be browsed by deer and regeneration is supported in gaps under the current deer density (10–13.5 deer km^−2^). Despite the similar deer density in both studies, the reason for the discrepancy remains unclear and may be an effect of diet preference between local deer populations. Nevertheless, our results indicate the possibility that treatment G could solve the declining *A. firma* sapling recruitment rates within the Boso peninsula (Abrams et al., 2017). On the other hand, *E. japonica* saplings showed higher recruitment and growth under increased light availability (Manabe & Yamamoto, 1997) and in the absence of deer (Tsujino & Yumoto, 2004). However, the increased sapling recruitment in the absence of deer for our study was only observed in the clearcut plots. This differed from the results of Tsujino & Yumoto (2004) where sapling recruitment were reported in both clearcut and closed canopy conditions. The reason for this discrepancy remains unclear but could be attributed to increased competition and limited light availability under a closed canopy in our study sites. Also, *E. japonica* saplings in treatment G were limited to less than 30 cm height while such limitations were not observed in treatment EG, which was expected given their palatability to deer (Agetsuma, Agetsuma-Yanagihara & Takafumi, 2011; Hashimoto & Fujiki, 2014). Thus, although *E. japonica* saplings are present in clearcut plots with deer present, successful regeneration to mature trees was suppressed under the current deer density.

Interestingly, *N. sericea* responded to deer differently under closed canopy and clearcut conditions, showing tolerance to low light intensity. The species is relatively unpalatable to deer (Tsujino & Yumoto, 2004) and can tolerate moderate deer densities under a closed canopy (22.8 deer km^−2^, Suda, Araki & Maruyama, 2001). Our results support those of Tsujino & Yumoto (2004) with higher sapling densities in treatment E compared to Ctrl but contradicts those of Suda, Araki & Maruyama (2001), as saplings were not detected in Ctrl plots. In contrast, under clearcut conditions, *N. sericea* sapling densities was greater with deer presence (G >EG), probably due to reduced competition by deer herbivory on other fast growing palatable tree species. The absence of this species in Ctrl plots suggests a lack of tolerance to current deer density under low light intensity, but sapling recruitment can be achieved through deer exclusion and improving light availability.

## Conclusion

The results of the 11-year experiment indicates that: (1) deer presence after clearcut delayed tree regeneration while increasing abundance of non-tree species, altering the composition of tree species from the original vegetation; (2) non-tree species exhibited negative relationship with tree cover in clearcut plots but strong inhibitory effects were only detected in the presence of deer; (3) recruitment of tree saplings was inhibited at the current deer density but this effect was not equal as the recruitment success depended on the species identity. In accordance with the need for more effective forest restoration (Castro et al., 2021), we recommend considering how current environmental conditions influence the recruitment success of each tree species. Knowledge on various responses of tree species to herbivory, gap creation and their combined effects may influence the decision of different implementation strategies needed to assist in forest regeneration.

##  Supplemental Information

10.7717/peerj.14210/supp-1 Supplemental Information 1 The locations of the study sites with visual representations of the experimental design and sampling within the study plots (A) The three study sites within the Boso peninsula, Hinokio (HNK) and Hiratsuka (HRT) and Kotsubosawa (KBS) are in warm temperate evergreen secondary forests that were previously coppiced for fuelwood before abandonment in the 1960′s (Suzuki, 2013). The sites are 2–5 km apart from each other located on hill slopes with angle of elevations between 28–36 degrees and elevations of 300 m above sea level (a.s.l). The map of Japan was downloaded from an online free resource (https://d-maps.com/carte.php?num_car=365&lang=en) while the map of Kamogawa city was generated using shapefiles downloaded from GADM (Select Japan from the drop down menu) and modified in ArcGisPro(Ver 2.5.2): https://gadm.org/download_country_v3.html.
(B) The 20-by-20 m plots at each study site were separated according to two levels of canopy cover (closed canopy and clearcut), each further divided into four 10-by-10 m subplots subjected to one of four treatment groups: **Control (Ctrl)** = Closed canopy and open to herbivores, **E** = Closed canopy with herbivores excluded, **G** = Clearcut and open to herbivores and **EG** = Clearcut with herbivores excluded.
(C) Vegetation surveys were conducted within 5 m quadrats established at the centre of each 10 m subplot to avoid boundary effects, between October and early November in 2019. The foliar cover of each plant species was visually estimated by dividing the 5 m quadrat into 100 units taking into account those in the ground and canopy layer. Sapling analyses of target common tree species was conducted according to the following height classes: (i) between 0.1 m to less than 0.3 m, (ii) between 0.3 m to less than 1 m, (iii) between 1 m to 2 m and (iv) higher than 2 m. Height classes (i) and (ii) were sampled within the 5-by-1 m belt transect at the centre of each subplot. Class (iii) and class (iv) saplings were sampled within 5-by-5 m quadrats and 10-by-10 m subplots respectively.

10.7717/peerj.14210/supp-2 Supplemental Information 2 Pictures of treatment plots in site HNK indicating the state of vegetation regeneration in 2019 The abbreviations Ctrl, G, E and EG refer to the “Closed canopy with deer” (Control), “Clearcut with deer”, “Closed canopy without deer” and “Clearcut without deer” treatments respectively.

10.7717/peerj.14210/supp-3 Supplemental Information 3 (A) Comparison of regression models in predicting non-tree cover and deer effects on tree foliar cover after clearcut. (B) Effects of non-tree species cover (%) and deer presence on tree foliar cover (%) in clearcut plots (G and EG) estimated from (A) ** indicates a significant deviation between the expected and observed values. NS indicates no significant deviation detected. Based on DHARMa residual diagnostics, only models M_0_ and M_2_ indicated that predicted values from the model did not deviate significantly from observed values. Based on the AIC and BIC values, we determined the best-fit model to be M_2_.
(B) Significant relationship are denoted by an asterisk.

10.7717/peerj.14210/supp-4 Supplemental Information 4 Mean (M) and standard deviation (SD) of sapling densities (m^-2^) of common tree species within each treatment group in 2020 Height class: (i) 10 cm ≤ ×  > 30 cm (ii) 30 cm ≤ ×  > 1 m (iii) 1 m ≤ ×  ≤ 2 m and (iv) > 2 m. Significant differences in sapling abundance within each height class among treatments is denoted by an asterisk.

10.7717/peerj.14210/supp-5 Supplemental Information 5 Total sapling density (m^−2^) of target tree species in 2020 The sapling density per height class (i to iv) of each species was calculated as (sapling count / sampled area m^−2^) for each subplot. These density values for each height class were then summed according to species and treatment to obtain the sapling density of each species per treatment group (*n* = 6). The total represents the sum of each species density among the treatment groups in the study. Significant differences in sapling density of each species among treatment groups following *post hoc* tests are displayed as different letters in superscript.

10.7717/peerj.14210/supp-6 Supplemental Information 6 Sapling (DBH > 10 cm) counts of selected tree species according to assigned height classes

10.7717/peerj.14210/supp-7 Supplemental Information 7 Plant species and percent cover in study plots The vegetation cover (%) of each plant species was assessed within 5-by-5 meter plots for each treatment group in 2019. Plants with cover less than 1% was recorded as ’+’ and assigned a value of 0.1 during statistical analyses.

10.7717/peerj.14210/supp-8 Supplemental Information 8 Calculated total tree sapling density data per study plot

10.7717/peerj.14210/supp-9 Supplemental Information 9 Tree species richness count per study plot

10.7717/peerj.14210/supp-10 Supplemental Information 10 Vegetation cover data for each study plot The above data is the sum of plant form cover expressed as percentage ratio within the 5 m vegetation survey quadrat of each study plot. A high overlap in plant cover results in values exceeding 1 which have been expressed as 0.999 for statistcal analysis (beta regression).

10.7717/peerj.14210/supp-11 Supplemental Information 11 R codes for Table 1

10.7717/peerj.14210/supp-12 Supplemental Information 12 R codes for Fig. 1

10.7717/peerj.14210/supp-13 Supplemental Information 13 R codes for Fig. 3 Model selection and GLMM

10.7717/peerj.14210/supp-14 Supplemental Information 14 R codes for Fig. 4 Non-parametric comparison of tree sapling abundance among treatment groups for each respective height class (i to iv).

10.7717/peerj.14210/supp-15 Supplemental Information 15 R codes for non-parametric comparison of total sapling density (n) among treatment groups for each target tree species

## Additional Information and Declarations

### Data Availability

The following information was supplied regarding data availability: The raw data and code are available in the Supplemental Files.

## References

[ref-1] Abrams MD, Umeki K, Bouma C, Nabeshima E, Toyama K (2017). A Dendroecological analysis of forest dynamics for old-growth abies-tsuga-quercus on the Boso Peninsula, Southeastern Japan. Tree-Ring Research.

[ref-2] Agetsuma N, Agetsuma-Yanagihara Y, Takafumi H (2011). Food habits of Japanese deer in an evergreen forest: litter-feeding deer. Mammalian Biology.

[ref-3] Castro J, Morales-Rueda F, Navarro FB, Löf M, Vacchiano G, Alcaraz-Segura D (2021). Precision restoration: a necessary approach to foster forest recovery in the 21st century. Restoration Ecology.

[ref-4] Chiba Prefecture of Japan (2004). Science report on the management of Sika Deer on the Boso Peninsula.

[ref-5] Chiba Prefecture of Japan (2017). Fourth science report on the management of sika deer on the Boso Peninsula.

[ref-6] Coomes DA, Allen RB, Forsyth DM, Lee WG (2003). Factors preventing the recovery of New Zealand forests following control of invasive deer. Conservation Biology.

[ref-7] Côté SD, Rooney TP, Tremblay JP, Dussault C, Waller DM (2004). Ecological impacts of deer overabundance. Annual Review of Ecology, Evolution, and Systematics.

[ref-8] Gill RMA (1992). A review of damage by mammals in north temperate forests: 1. Deer. Forestry: An International Journal of Forest Research.

[ref-9] Hashimoto Y, Fujiki D (2014). List of food plants and unpalatable plants of sika deer (*Cervus nippon*) in Japan. Humans and Nature.

[ref-10] Hisamoto Y, Oishi S, Suzuki M, Tsurumi Y, Yonemichi T, Suzuki M (2019). Efficiency of estimating sika deer density using camera traps in the University of Tokyo Chiba Forest (in Japanese). Miscellaneous Information of the University of Tokyo Forests.

[ref-11] Kern CC, Reich PB, Montgomery RA, Strong TF (2012). Do deer and shrubs override canopy gap size effects on growth and survival of yellow birch, northern red oak, eastern white pine, and eastern hemlock seedlings?. Forest Ecology and Management.

[ref-12] Koda R, Noma N, Tsujino R, Umeki K, Fujita N (2008). Effects of sika deer (*Cervus nippon yakushimae*) population growth on saplings in an evergreen broad-leaved forest. Forest Ecology and Management.

[ref-13] Manabe T, Yamamoto SI (1997). Spatial distribution of Eurya japonica in an old-growth evergreen broad-leaved forest, SW Japan. Journal of Vegetation Science.

[ref-14] Nagashima K, Shimomura T, Tanaka K (2019). Early-stage vegetation recovery in forests damaged by oak wilt disease and deer browsing: effects of deer-proof fencing and clear-cutting. LandScape and Ecological Engineering.

[ref-15] Petersson LK, Dey DC, Felton AM, Gardiner ES, Löf M (2020). Influence of canopy openness, ungulate exclosure, and low-intensity fire for improved oak regeneration in temperate Europe. Ecology and Evolution.

[ref-16] R Core Team (2021). https://www.R-project.org/.

[ref-17] Royo AA, Carson WP (2006). On the formation of dense understory layers in forests worldwide: consequences and implications for forest dynamics, biodiversity, and succession. Canadian Journal of Forest Research.

[ref-18] Sabo AE, Forrester JA, Burton JI, Jones PD, Mladenoff DJ, Kruger EL (2019). Ungulate exclusion accentuates increases in woody species richness and abundance with canopy gap creation in a temperate hardwood forest. Forest Ecology and Management.

[ref-19] Shimoda K, Kimura K, Kanzaki M, Yoda K (1994). The regeneration of pioneer tree species under browsing pressure of Sika deer in an evergreen oak forest. Ecological Research.

[ref-20] Suda K, Araki R, Maruyama N (2001). The effects of sika deer on the structure and composition of the forests on the Tsushima islands. Biosphere Conservation: For Nature, Wildlife, and Humans.

[ref-21] Suzuki M (2013). Succession of abandoned coppice woodlands weakens tolerance of ground-layer vegetation to ungulate herbivory: a test involving a field experiment. Forest Ecology and Management.

[ref-22] Suzuki M, Ikeda H, Karukome T, Fujihira K, Tsukagoshi T, Mitsugi M, Satomi S, Adachi Y, Murakawa I, Otsuka A, Hiroshima T, Yamanaka I, Yamada T (2010). A long-term field experiment on the tree regeneration and ecosystem restoration of secondary broad-leaved forests with overabundant deer: the 12th management and experiment plan of the Tokyo University Forest in Chiba. Miscellaneous Information, the University of Tokyo Forests.

[ref-23] Suzuki M, Ito E (2014). Combined effects of gap creation and deer exclusion on restoration of belowground systems of secondary woodlands: a field experiment in warm-temperate monsoon Asia. Forest Ecology and Management.

[ref-24] Suzuki M, Karukome T, Fujihira K, Mitsugi M, Hisatmoto Y (2021). Clearcutting triggers regeneration of abandoned secondary forests but has a risk of alternative successional trajectory with high deer density. Applied Vegetation Science.

[ref-25] Suzuki M, Miyashita T, Kabaya H, Ochiai K, Asada M, Tange T (2008). Deer density affects ground-layer vegetation differently in conifer plantations and hardwood forests on the Boso Peninsula, Japan. Ecological Research.

[ref-26] Takatsuki S (2009). Effects of sika deer on vegetation in Japan: a review. Biological Conservation.

[ref-27] Takatsuki S, Hirabuki Y (1997). Effects of Sika Deer browsing on the structure and regeneration of the *Abies firma* Forest on Kinkazan Island, Northern Japan. Journal of Sustainable Forestry.

[ref-28] Tamura A, Nakajima K (2017). Effects of 10 years of fencing under a gap and closed canopy on the regeneration of tree seedlings in an old-growth Japanese fir (*Abies firma*) forest overbrowsed by sika deer. Journal of Forest Research.

[ref-29] The University of Tokyo Forests (2021). Annual Report of Meteorological Observations in the University Forests, The University of Tokyo (Jan 2019 - Dec. 2019). Miscellaneous Information of the University of Tokyo Forests.

[ref-30] Tremblay JP, Huot J, Potvin F (2007). Density-related effects of deer browsing on the regeneration dynamics of boreal forests. Journal of Applied Ecology.

[ref-31] Tsujino R, Yumoto T (2004). Effects of sika deer on tree seedlings in a warm temperate forest on Yakushima Island, Japan. Ecological Research.

[ref-32] Yamamoto SI (2000). Forest gap dynamics and tree regeneration. Journal of Forest Research.

