# Supplementary figures and images for: Regeneration of tree species after 11 years of canopy gap creation and deer exclusion in a warm temperate broad-leaved forest over-browsed by sika deer
Source: PeerJ. 2022 Nov 1;10:e14210. doi: 10.7717/peerj.14210 (PMC9635360; doi:10.7717/peerj.14210)

Supplementary file A1

Study sites


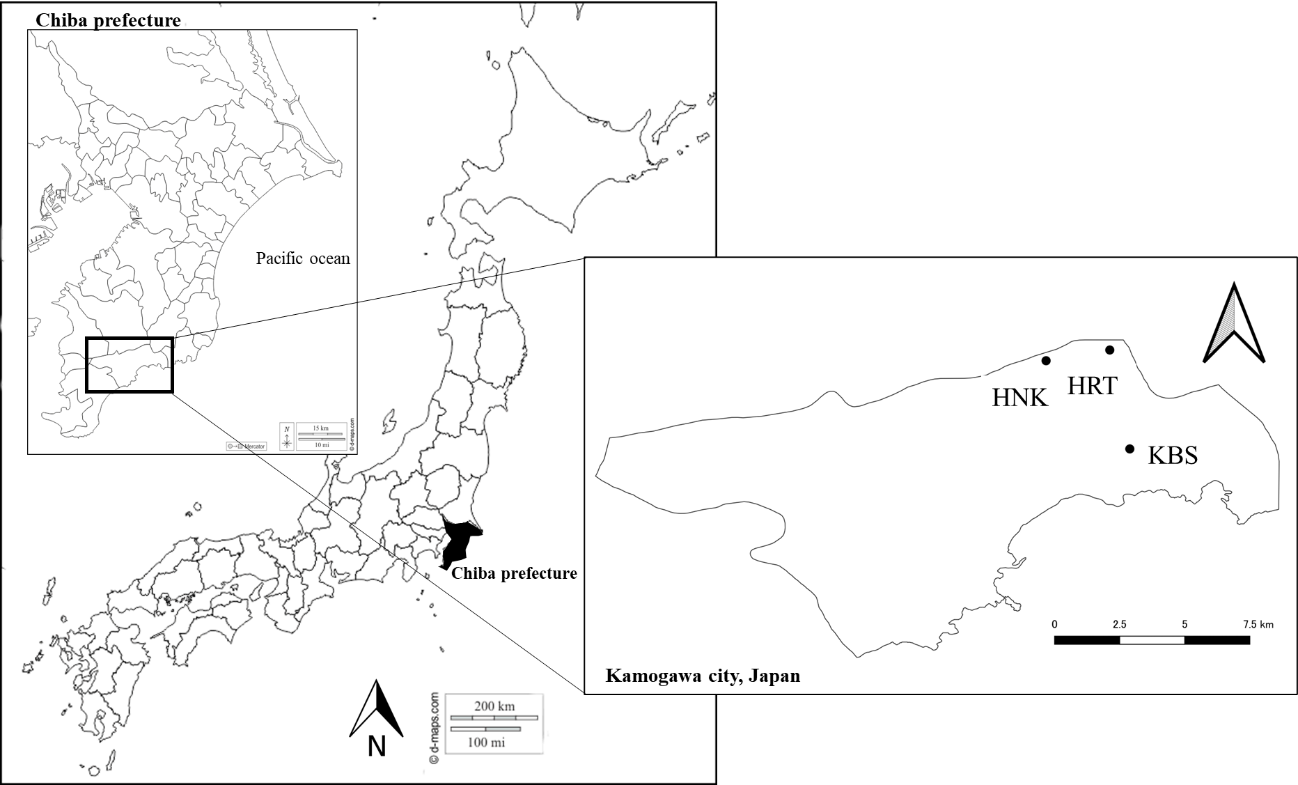


**B**

Experimental Design


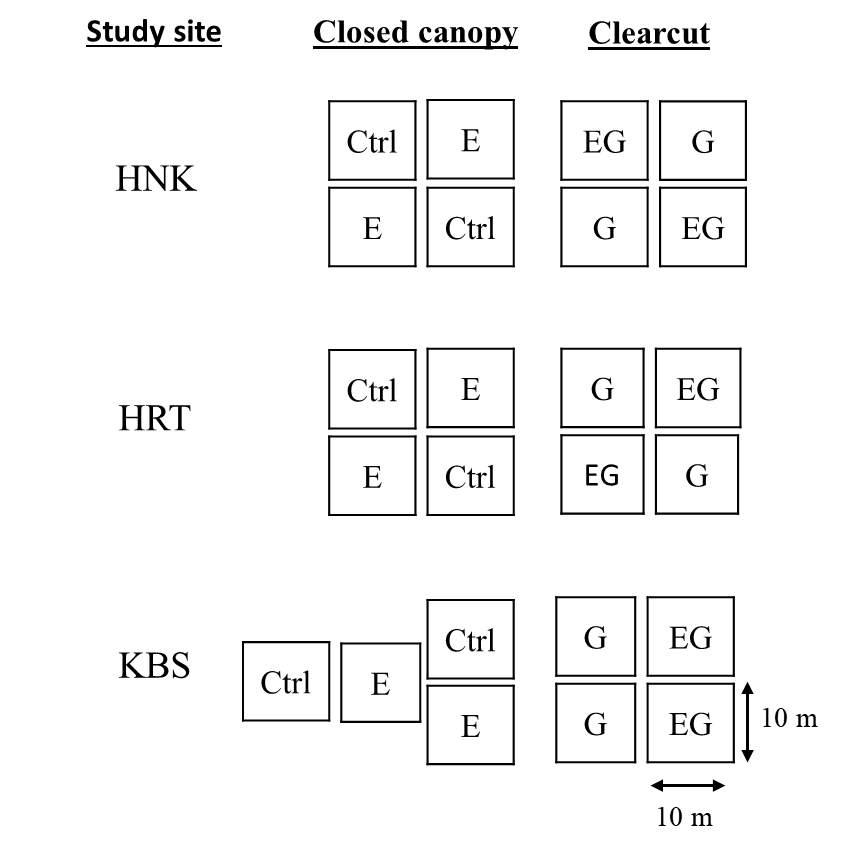


**C.**

Vegetation and Sapling surveys


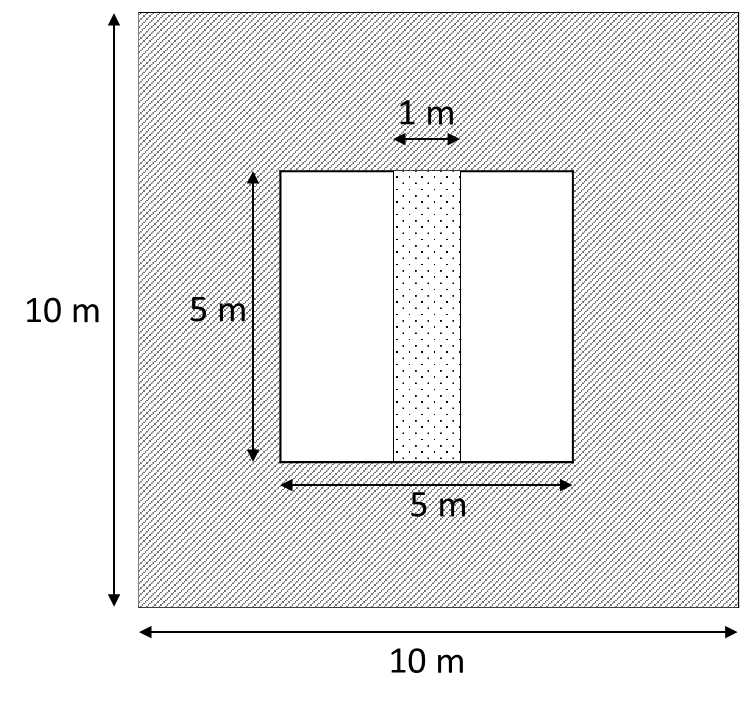

Supplement: Supplemental Information 1 — (A) The three study sites within the Boso peninsula, Hinokio (HNK) and Hiratsuka (HRT) and Kotsubosawa (KBS) are in warm temperate evergreen secondary forests that were previously coppiced for fuelwood before abandonment in the 1960′s (Suzuki, 2013). The sites are 2–5 km apart from each other located on hill slopes with angle of elevations between 28–36 degrees and elevations of 300 m above sea level (a.s.l). The map of Japan was downloaded from an online free resource (https://d-maps.com/carte.php?num_car=365&lang=en) while the map of Kamogawa city was generated using shapefiles downloaded from GADM (Select Japan from the drop down menu) and modified in ArcGisPro(Ver 2.5.2): https://gadm.org/download_country_v3.html. (B) The 20-by-20 m plots at each study site were separated according to two levels of canopy cover (closed canopy and clearcut), each further divided into four 10-by-10 m subplots subjected to one of four treatment groups: Control (Ctrl) = Closed canopy and open to herbivores, E = Closed canopy with herbivores excluded, G = Clearcut and open to herbivores and EG = Clearcut with herbivores excluded. (C) Vegetation surveys were conducted within 5 m quadrats established at the centre of each 10 m subplot to avoid boundary effects, between October and early November in 2019. The foliar cover of each plant species was visually estimated by dividing the 5 m quadrat into 100 units taking into account those in the ground and canopy layer. Sapling analyses of target common tree species was conducted according to the following height classes: (i) between 0.1 m to less than 0.3 m, (ii) between 0.3 m to less than 1 m, (iii) between 1 m to 2 m and (iv) higher than 2 m. Height classes (i) and (ii) were sampled within the 5-by-1 m belt transect at the centre of each subplot. Class (iii) and class (iv) saplings were sampled within 5-by-5 m quadrats and 10-by-10 m subplots respectively. [file peerj-10-14210-s001.docx]

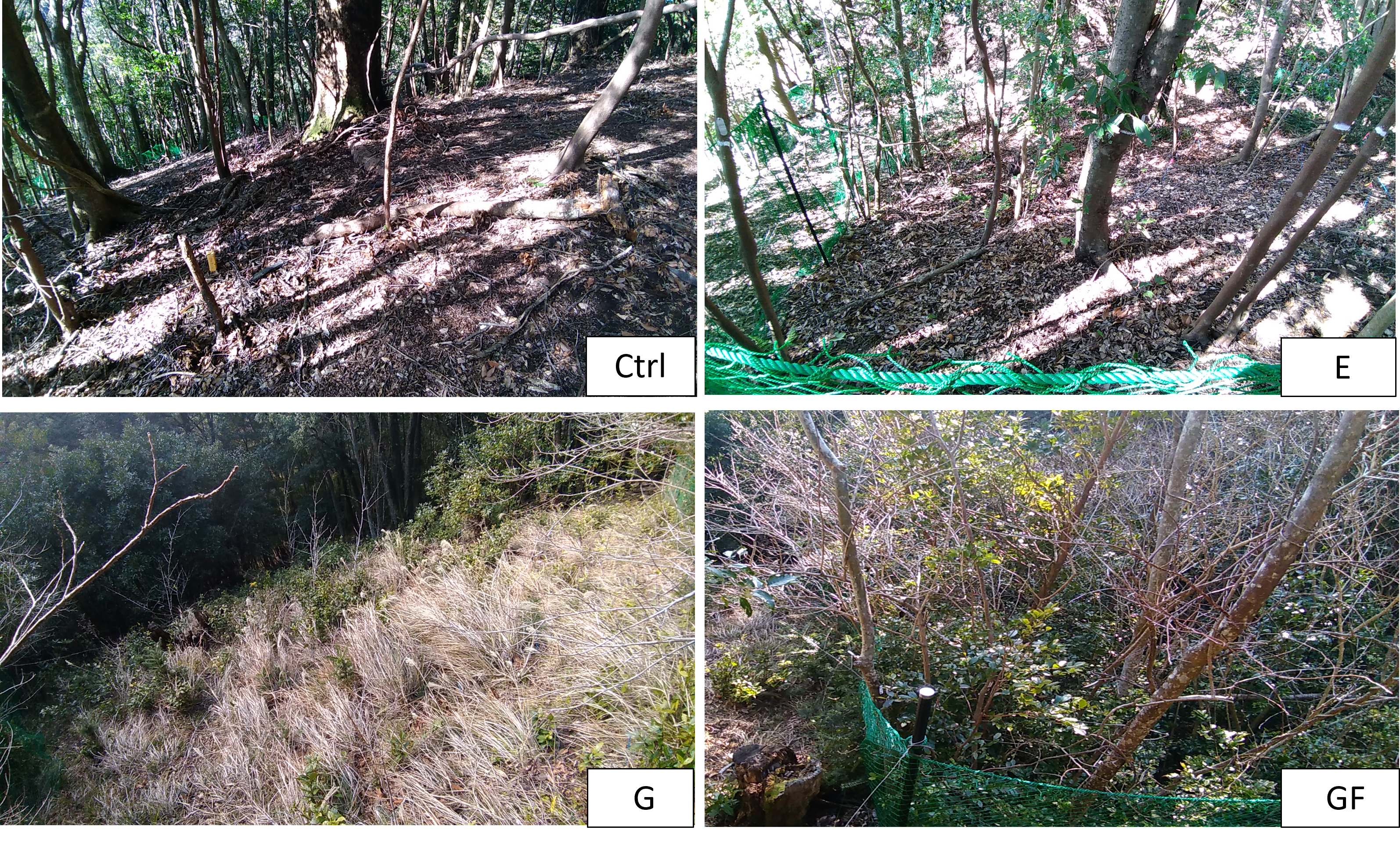

Supplement: Supplemental Information 2 — The abbreviations Ctrl, G, E and EG refer to the “Closed canopy with deer” (Control), “Clearcut with deer”, “Closed canopy without deer” and “Clearcut without deer” treatments respectively. [file peerj-10-14210-s002.png]
