# Supplementary material for: Regeneration of tree species after 11 years of canopy gap creation and deer exclusion in a warm temperate broad-leaved forest over-browsed by sika deer
Source: PeerJ. 2022 Nov 1;10:e14210. doi: 10.7717/peerj.14210 (PMC9635360; doi:10.7717/peerj.14210)
Supplement: Supplemental Information 3 — (A) ** indicates a significant deviation between the expected and observed values. NS indicates no significant deviation detected. Based on DHARMa residual diagnostics, only models M0 and M2 indicated that predicted values from the model did not deviate significantly from observed values. Based on the AIC and BIC values, we determined the best-fit model to be M2. (B) Significant relationship are denoted by an asterisk. [file peerj-10-14210-s003.docx]

Supplementary file A3.

**A**

| Model | Description |  | AIC | BIC | DHARMa residual diagnostics |
| --- | --- | --- | --- | --- | --- |
| M_0_ | non-tree cover + Deer + non-tree cover*Deer + (1\| location/plot) |  | -2.6 | 0.8 | NS |
| M_1_ | non-tree cover + Deer + (1\| location/plot) |  | -3.0 | -0.05 | ** |
| M_2_ | non-tree cover + Deer + (1\| plot) |  | -5.0 | -2.5 | NS |
| M_3_ | non-tree cover + Deer + non-tree cover*Deer + (1\| plot) |  | -4.6 | -1.7 | ** |

**B**

| Fixed variable |  | Estimate | SE | p |
| --- | --- | --- | --- | --- |
| Intercept (Tree cover) |  | 1.86 | 0.50 |  |
| Non-tree species cover (%) |  | -2.05 | 1.33 | 0.12 |
| Deer presence (0 or 1) |  | -2.12 | 0.56 | <0.01* |
