# Supplementary material for: Regeneration of tree species after 11 years of canopy gap creation and deer exclusion in a warm temperate broad-leaved forest over-browsed by sika deer
Source: PeerJ. 2022 Nov 1;10:e14210. doi: 10.7717/peerj.14210 (PMC9635360; doi:10.7717/peerj.14210)
Supplement: Supplemental Information 4 — Height class: (i) 10 cm ≤ × > 30 cm (ii) 30 cm ≤ × > 1 m (iii) 1 m ≤ × ≤ 2 m and (iv) > 2 m. Significant differences in sapling abundance within each height class among treatments is denoted by an asterisk. [file peerj-10-14210-s004.docx]

| Treatment | Height | Area (m^2^) | *Abies firma* | | *Neolitsea sericea* | | *Eurya japonica* | | *Cinnamomum tenuifolium* | | *Quercus acuta* | | *Castanopsis sieboldii* | |
| --- | --- | --- | --- | --- | --- | --- | --- | --- | --- | --- | --- | --- | --- | --- |
|  | class |  | M | SD | M | SD | M | SD | M | SD | M | SD | M | SD |
| Ctrl | i | 30 |  |  |  |  | 0.43 | 0.61 |  |  |  |  |  |  |
|  | ii | 30 |  |  |  |  | 0.17 | 0.27 |  |  |  |  |  |  |
|  | iii | 150 |  |  | 0.01 | 0.02 |  |  |  |  |  |  |  |  |
|  | iv | 600 |  |  |  |  |  |  |  |  |  |  |  |  |
| E | i |  |  |  | 0.1 | 0.17 |  |  | 0.07 | 0.1 | 0.03 | 0.08 | 0.07 | 0.1 |
|  | ii |  |  |  | 0.03 | 0.08 |  |  | 0.03 | 0.08 | 0.3 | 0.5 | 0.2 | 0.33 |
|  | iii |  |  |  | 0.01 | 0.02 | 0.03 | 0.06 | 0.03 | 0.06 | 0.03 | 0.05 | 0.08 | 0.13 |
|  | iv |  |  |  |  |  |  |  |  |  |  |  |  |  |
|  |  |  |  |  |  |  |  |  |  |  |  |  |  |  |
| G | i |  | 0.43 | 0.87 |  |  | 1.53*^(G-E)^ | 1.51 |  |  |  |  |  |  |
|  | ii |  | 0.2 | 0.25 |  |  | 0.27 | 0.24 |  |  |  |  |  |  |
|  | iii |  | 0.02 | 0.03 | 0.07 | 0.09 |  |  |  |  |  |  |  |  |
|  | iv |  |  |  | 0.11 | 0.17 |  |  |  |  |  |  |  |  |
| EG | i |  | 0.17 | 0.32 |  |  | 0.73 | 0.86 |  |  | 0.07 | 0.16 |  |  |
|  | ii |  | 0.17 | 0.41 |  |  | 3* | 1.92 |  |  | 0.3 | 0.41 | 0.03 | 0.08 |
|  | iii |  | 0.01 | 0.02 | 0.01 | 0.02 | 1.15* | 0.93 | 0.04 | 0.06 | 0.09 | 0.12 | 0.01 | 0.03 |
|  | iv |  |  |  | 0.02 | 0.03 | 0.39* | 0.29 | 0.06* | 0.04 | 0.34* | 0.35 | 0.14* | 0.11 |
