# Supplementary material for: Regeneration of tree species after 11 years of canopy gap creation and deer exclusion in a warm temperate broad-leaved forest over-browsed by sika deer
Source: PeerJ. 2022 Nov 1;10:e14210. doi: 10.7717/peerj.14210 (PMC9635360; doi:10.7717/peerj.14210)
Supplement: Supplemental Information 5 — The sapling density per height class (i to iv) of each species was calculated as (sapling count / sampled area m−2) for each subplot. These density values for each height class were then summed according to species and treatment to obtain the sapling density of each species per treatment group (n = 6). The total represents the sum of each species density among the treatment groups in the study. Significant differences in sapling density of each species among treatment groups following post hoc tests are displayed as different letters in superscript. [file peerj-10-14210-s005.docx]

| Species name | Ctrl | E | G | EG | Total |
| --- | --- | --- | --- | --- | --- |
|  | n = 6 | n = 6 | n = 6 | n = 6 |  |
| *Abies firma* |  |  | 3.92 | 2.04 | 5.96 |
| *Neolitsea sericea* |  | 0.84 | 1.06 | 0.22 | 2.16 |
| *Eurya japonica* | 3.60^a^ | 0.20^a^ | 10.80^a^ | 31.6^b^ | 46.20 |
| *Castanopsis sieboldii* | 0^a^ | 2.08^b^ | 0^a^ | 1.12^ab^ | 3.20 |
| *Cinnamomum tenuifolium* | 0^a^ | 0.80^b^ | 0.01^a^ | 0.61^ab^ | 1.42 |
| *Quercus acuta* | 0^a^ | 2.16^a^ | 0^a^ | 4.77^b^ | 6.93 |
